# MRI in Early Onset of Creutzfeldt Jakob Disease

**DOI:** 10.5334/jbsr.2352

**Published:** 2021-06-08

**Authors:** Marie Lorent, Wim Verwimp, Stephane Dechambre

**Affiliations:** 1 Centre hospitalier de Mouscron, BE

**Keywords:** creutzfeldt Jakob, MRI, DWI, dementia

## Abstract

**Teaching point:** Cortical diffusion restriction can be depicted by MRI in the early onset of Creutzfeld Jakob disease.

## Case

A 79-year-old woman presented a rapidly progressive dementia, headaches, retrograde amnesia of recent memories, and spatio-temporal disorientation. Symptoms started two months earlier while neurological examination was normal. The electroencephalogram showed slow cycles with no periodic sharp wave complexes.

**Figure 1:**
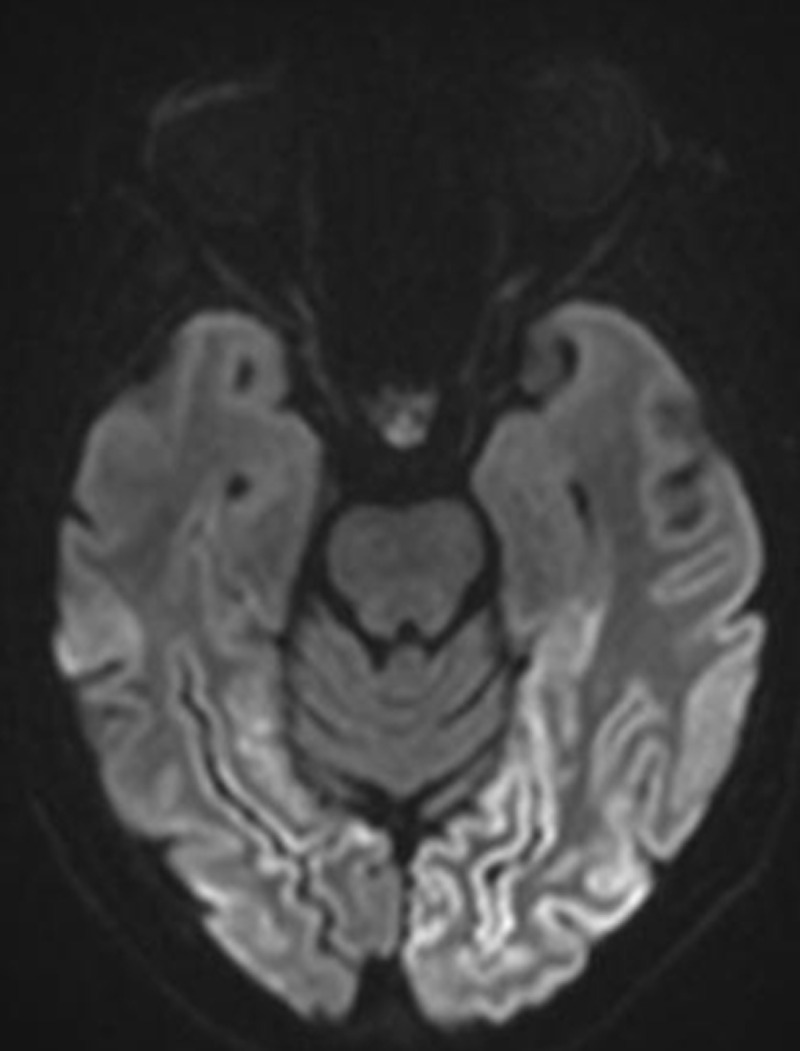


**Figure 2:**
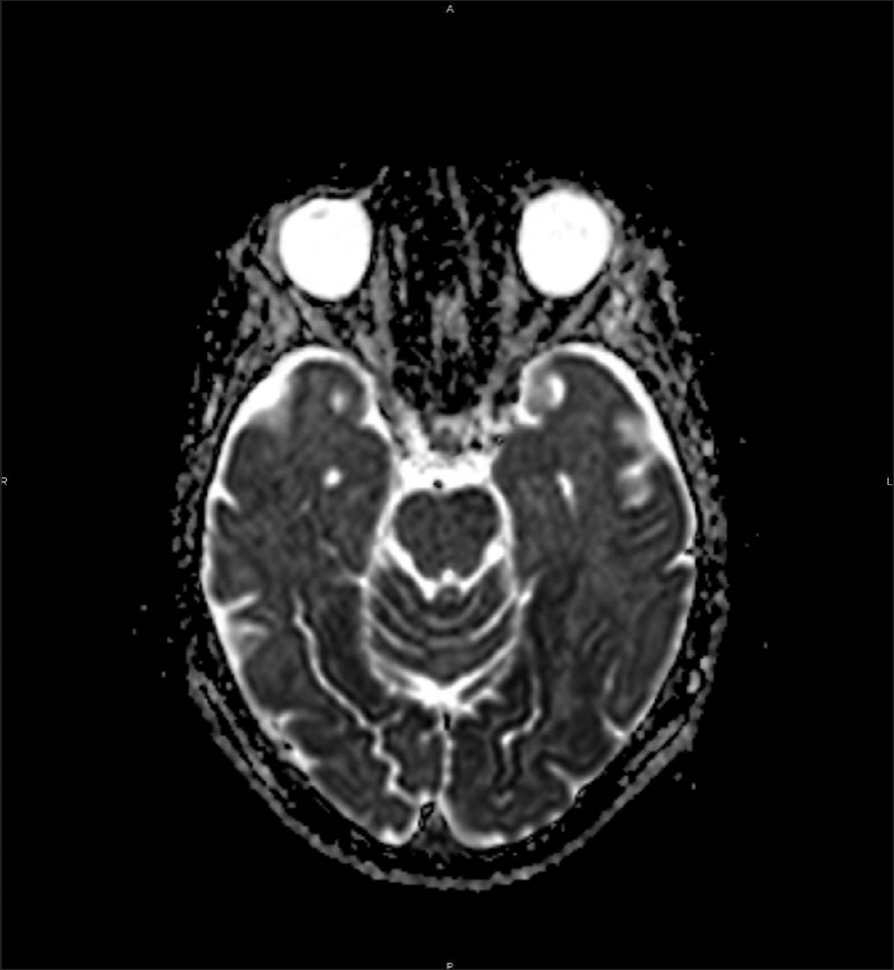


**Figure 3:**
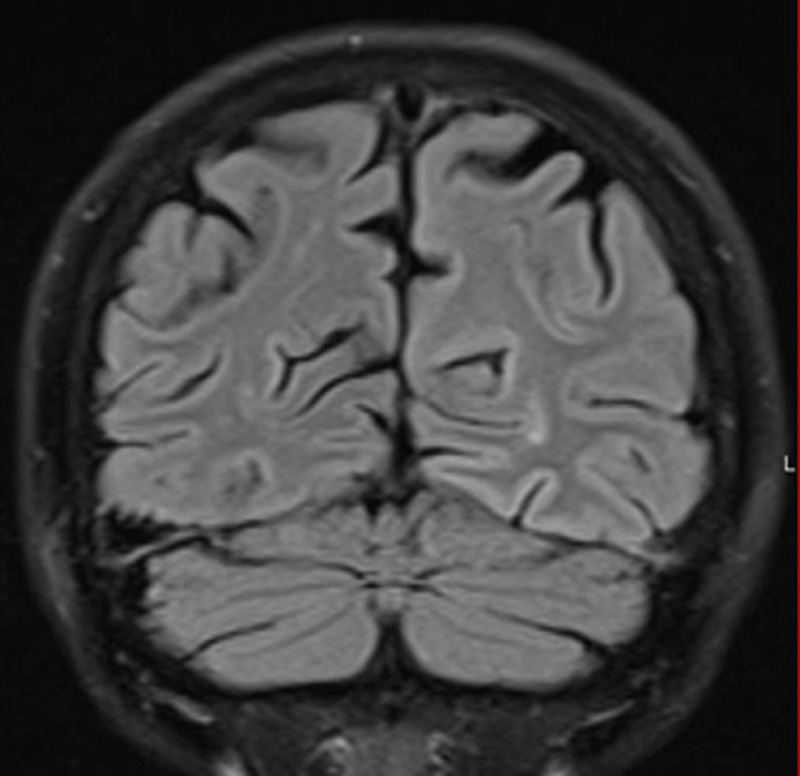


Diffusion-weighted (DW) magnetic resonance imaging (MRI) revealed bilateral widespread cortical diffusion restriction with occipital and temporal topography (***Figure 1***). A decrease of the apparent diffusion coefficient (***Figure 2***) and minimal signal abnormality on fluid attenuated inversion recovery images were also observed in the affected areas (***Figure 3***).

At the time of MRI, 14-3-3 protein, a characteristic and suggestive biomarker for Creutzfeldt Jakob disease, was negative. Fifteen days later a second analysis was positive.

Patient follow-up showed a rapid decrease of the mental state, language disabilities, and cerebellar symptoms. She died two months later.

## Comment

Creutzfeld Jakob is a transmissible neurodegenerative disorder caused by prions which accumulate in and around neurons leading to cell death. The probable diagnosis relies on the combination of clinical features, electroencephalogram, cerebrospinal fluid analysis, and MRI findings, which are characterized by a diffusion restriction attributed to spongiform neuronal degeneration [1]. Other T2-weighted imaging sequences are less sensitive. Typically, signal alterations involve the cortical areas, the pulvinar nuclei of the thalamus (hockey stick sign), and basal ganglia [1]. The limbic lobe can also be involved and the perirolandic area is often spared [1]. In the presence of cerebellar symptoms, infratentorial DW imaging is usually negative; however, cerebellar atrophy is common.

At early stage of the disease, only the cortical areas can be affected.

During disease progression, MRI signal abnormalities can decrease and even disappear [1].
